# Diversity of ticks and tick-borne pathogens in ticks removed from dogs and cats: a focus on Poland, Czech Republic, Slovakia, Hungary, and Romania

**DOI:** 10.1186/s13071-025-06852-6

**Published:** 2025-07-21

**Authors:** Sajjad Ghodrati, Andra Nica, Michal Ceregrzyn, Lukasz Adaszek, Jan Doležal, Gianluca D’Amico, David Modrý

**Affiliations:** 1 Department of Botany and Zoology, Faculty of Science, Masaryk University, Brno, Czech Republic; 2 Department of Veterinary Sciences, Faculty of Agrobiology, Food and Natural Resources, Czech University of Life Sciences, Prague, Czech Republic; 3 Biology Centre, Institute of Parasitology, Czech Academy of Sciences, České Budějovice, Czech Republic; 4 MSD Animal Health, Rahway, USA; 5 Department of Epizootiology and Clinic of Infectious Diseases, University of Life Sciences, Lublin, Poland; 6 Department of Parasitology and Parasitic Diseases, University of Agricultural Sciences and Veterinary Medicine Cluj-Napoca, Cluj-Napoca-Napoca, Romania

**Keywords:** Ticks, *Ixodes ricinus*, *Dermacentor reticulatus*, *Ixodes hexagonus*, Companion animals, Dog, Cat, Vector-borne, Central European Region

## Abstract

**Background:**

Ticks in Europe comprise over 100 species, with *Ixodes*, *Rhipicephalus*, *Dermacentor*, and *Haemaphysalis* being the most prevalent. *Ixodes ricinus* is the most widespread, while *Dermacentor reticulatus* is common in central Europe. *Rhipicephalus sanguineus* has expanded into temperate regions, affecting tick–host interactions and pathogen transmission. Companion animals, especially dogs and cats, are common tick hosts and vectors for pathogens such as borreliosis, anaplasmosis, and canine babesiosis. This study investigates the prevalence and species composition of ticks infesting dogs and cats in Poland, the Czech Republic, Slovakia, Hungary, and Romania, focusing on seasonal trends and pathogen occurrence.

**Methods:**

From 2020 to 2022, ticks were collected from dogs and cats during veterinary consultations across five countries. A total of 4563 ticks were identified morphologically, and 1085 were screened for pathogens, including *Babesia canis*, *Anaplasma phagocytophilum*, *Ehrlichia canis*, and *Borrelia burgdorferi* sensu lato.

**Results:**

*I. ricinus* was the predominant species (65.0%), followed by *D. reticulatus* (29.8%) and *R. sanguineus* (3.8%). Ticks were present year-round, with peaks in spring and autumn. Of the 1085 tested ticks, 29.0% were positive for at least one pathogen, with *A. phagocytophilum* being the most common (15.0%). *B. burgdorferi* s.l. was detected in 7.0% of ticks, and *Babesia* spp. in 6.5%, predominantly *B. canis*.

**Conclusions:**

This study highlights the year-round risk of tick infestations and vector-borne pathogen transmission in dogs and cats in Central Europe, underscoring the need for ongoing tick surveillance. Veterinary practitioners should enhance public awareness about ticks and associated health risks for companion animals.

**Graphical abstract:**

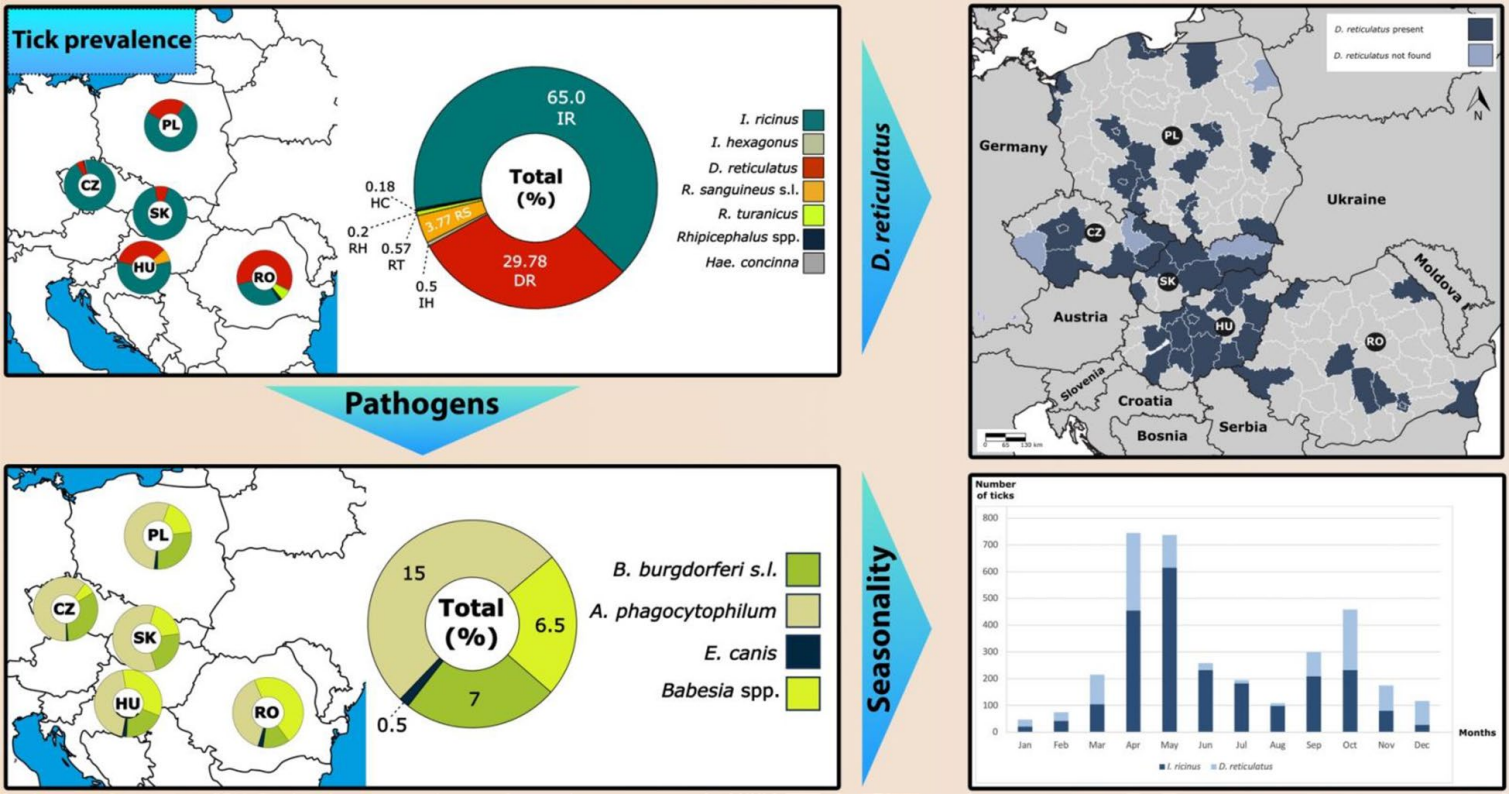

**Supplementary Information:**

The online version contains supplementary material available at 10.1186/s13071-025-06852-6.

## Background

In Europe, ticks are a highly diverse group, comprising over 100 species distributed across various genera [1]. The most prevalent genera include *Ixodes*, *Rhipicephalus*, *Dermacentor*, *Haemaphysalis*, and *Rhipicephalus*, with *Ixodes ricinus* being the predominant species in numerous regions [2]. The second most reported tick in Europe is *Dermacentor reticulatus*. While there is some overlap in the ranges of *I. ricinus* and *D. reticulatus*, the latter has a more restricted distribution, primarily found in central Europe [3]. In Europe, *Rhipicephalus sanguineus* sensu lato (s.l.) is considered to be mainly a Mediterranean tick species that can locally invade temperate regions. This is evidenced by the increasing number of discoveries in northern countries such as the UK, Ireland, Denmark, Germany, Hungary, and Sweden [4–6]. In recent years, Europe has experienced changes in tick distribution due to climate change, land use modifications, and increased animal mobility [7].

*R. sanguineus* s.l., traditionally considered a single species, has undergone taxonomic revisions, revealing multiple cryptic species within this group [8–11]. This taxonomic division is pivotal for comprehending the ecology and epidemiology of tick-borne diseases as distinct species within the *R. sanguineus* s.l. complex that may exhibit varying geographical distributions and host preferences [12].

Ticks in Europe exhibit diverse host associations, with some species being generalists and others specialists. For instance, *I. ricinus* is a generalist, feeding on a wide range of mammals, birds, and reptiles, while species such as *Ixodes hexagonus* specialize in parasitizing hedgehogs [1, 13]. Stage-specific preferences are prevalent, with larvae and nymphs feeding on small mammals and birds, and adults on larger mammals such as deer. These host associations are essential for tick survival and disease transmission [14]. Climate and habitat changes are reshaping these interactions across Europe [15]. Ticks that are commonly infesting dogs and cats are *R. sanguineus* s.l. (brown dog ticks), which is a vector for *Ehrlichia canis*, *Rickettsia conorii*, and *Babesia vogeli*. *I. ricinus* transmits etiological agents of Lyme disease and anaplasmosis. *D. reticulatus* is another significant species, primarily infesting dogs and spreading *Babesia canis* [7, 12, 16–18].

Ticks are significant vectors of pathogens, and their distribution directly influences the spread of tick-borne diseases [18]. For instance, *D. reticulatus* has expanded its range, introducing the tick-borne pathogen *B. canis* to new regions. This tick is the primary vector for *B. canis*, which causes canine babesiosis, a severe disease affecting dogs. The expanding distribution of *D. reticulatus* reflects the increasing incidence of *B. canis* in areas where the tick was previously rare, such as parts of Central and Northern Europe [19, 20].

Pathogen–host relationships in tick-borne diseases vary. Some pathogens, such as *Borrelia* spp., *A. phagocytophilum*, and tick-borne encephalitis virus (TBEV), have a broad host range, while others, such as *A. platys*, *Babesia* spp., and *Hepatozoon* spp., exhibit a more restricted host spectrum [21–24]. Advancements in diagnostics have challenged previously narrow vector associations, such as *Hepatozoon canis* being traditionally associated with *R. sanguineus*, but is now found across Europe despite the tick’s absence [16, 25].

Citizen science and veterinary practitioners play pivotal roles in tick research, significantly advancing our understanding of tick distribution, seasonality, and host associations [26]. Programs such as “Najdi pijáka” (https://najdipijaka.cz/) and “TickSpotters” (https://web.uri.edu/tickencounter/tickspotters/submit/) in the Czech Republic and the USA have utilized public reports to monitor the spread of *D. reticulatus* and *I. scapularis*, respectively [19, 27]. The example of a study utilizing the collection of ticks found on animals, referred to routine veterinary visits, allowed for analysis of tick species prevalence and seasonality of infestations [28].

The distribution of ticks that affect companion animals in Europe undergoes dynamic development, attributable to various causes such as climatic and environmental changes, and mobility of companion animals [29, 30]. As a reaction to this trend, our study investigates tick prevalence and species composition infesting dogs and cats presented to veterinarians during routine consultations at veterinary hospitals in Poland, the Czech Republic, Slovakia, Hungary, and Romania. Besides answering fundamental questions about cats as hosts for ticks, we aimed to characterize the seasonality and geographic differences in tick distribution, as well as the occurrence of major tick-borne pathogens in collected ticks.

## Methods

### Tick collection and identification

The research was multicentered and conducted between 2020 and 2022 as part of the “Protect Our Future Too” project. A total of 4563 ticks were collected at 74 veterinary clinics in five countries: Poland, Hungary, the Czech Republic, Slovakia, and Romania. The veterinary clinics were selected to cover a representative geographical area for the region and within each country. The final number of participating veterinary clinics was influenced by their willingness to cooperate. This varied between countries, which accounts for the different number of veterinary clinics involved in each country (Hungary: 18, Czech Republic: 21, Romania: 8, Slovakia: 13, and Poland: 14). Ticks were manually collected from dogs and cats during routine clinical consultations. Ticks were preserved in 70% ethanol; basic anamnestic data were marked in a sheet attached to each batch of ticks collected from an individual dog or cat and sent to the examination laboratory. All ticks were examined using a stereomicroscope and classified into stages (larva, nymph, adult male/female) and species were identified on the basis of the standard morphological keys [1, 31, 32]. A subset of different tick genera representatives was randomly selected and photographed under a KEYENCE VHX-5000 digital microscope (Keyence, Belgium). Molecular identification of *I. ricinus* and *I. inopinatus* ticks was not performed in this study. Therefore, all ticks morphologically resembling *I. ricinus* were classified as *I. ricinus*/*inopinatus* and named *I. ricinus* throughout the text. Subsequently, all the samples were subjected to DNA isolation using a commercial Qiagen DNA Mini Kit (Qiagen, Hilden, Germany) according to the manufacturer’s instructions. The DNA was stored at 4 ℃ until further processing.

### Pathogen detection and identification

A subset of 1085 random ticks (198 ticks from Romania, 114 ticks from Poland, 339 ticks from the Czech Republic, 108 ticks from Slovakia, and 326 ticks from Hungary) was subjected to molecular detection of pathogens, targeting the 18S rRNA of *B. canis*, and the 16S rRNA of *A. phagocytophilum*, *E. canis*, and *Borrelia burgdorferi* s.l. Before DNA isolation, the selected ticks were washed in 70% ethanol and sterile water, and homogenized in 100 μL of phosphate-buffered saline (PBS) with a sterile pestle. DNA was extracted from individual ticks using the Genomic Mini kit (A&A Biotechnology, Poland) according to the manufacturer’s instructions, preceded by a 6 h digestion with proteinase K. Following extractions, all DNA samples were labeled and amplified by real-time PCR using a Rotor-Gene thermocycler (Corbett Research, Mortlake, Australia). The list of primers used for all studied pathogens and the reaction conditions are presented in Table 1. The real-time PCR with SYBR Green 1 dye was carried out in thin-walled test tubes with a capacity of 100 μL. A DyNAmo HS SYBR Green qPCR Kit (Finnzymes, Espoo, Finland) was used to conduct a high-specificity reaction. The reaction mixture with a volume of 20 µL consisted of the following components: 2 µL of the DNA matrix, 0.4 µL of each primer, 10 µL of Master Mix containing a hot start version of the modified polymerase Tbr (*Thermus brockianus*), buffer for the polymerase Tbr, dNTP, MgCl_2_, the intercalating SYBR Green 1 dye, and water to 20 µL. For each PCR, a negative control containing PCR water and a positive control containing genomic DNA of the tested pathogen were included. The DNA of *B. burgdorferi* s.l., DNA of *A. phagocytophilum*, and DNA of *E. canis* was obtained from the National Reference Center for *Borreliae* of the Max von Pettenkofer Institute of Ludwig Maximilian University Munich. *Babesia* DNA, isolated from the blood of dogs infected with *B. canis*, were also used as positive controls. PCR products were purified using the QIAquick spin columns (Qiagen) and eluted in 50 mL of Tris–HCl buffer (10 mM, pH 7.6). The DNA sequence of *Babesia* was determined on both strands using the same primers employed for PCR at a DNA sequencing core facility (Research Institute, Polish Academy of Sciences, Warsaw, Poland). DNA sequences were assembled and edited using SeqMan (DNAstar, Lasergene, Madison, USA) and Clustal V alignments to the published *B. canis* 18S rRNA gene (GenBank accession numbers: EU 622793 and EU 622792) and *B. microti* 18S rRNA gene (GenBank accession numbers: MK609547, AB190459, and AB243679).

**Table 1:** List of primers used for the detection of tick-borne pathogens in this study

Pathogen	Primers	Target gene	Product length (bp)	Annealing (°C)	References
*E. canis*	EC1: (5′-TTA-TAGCCTCTGGCTATAGG-3′ EC2: (5′-CACTTTTAACTTACTAGTCC-3′)	16S rRNA	495	50	[80]
*Babesia* spp.	BAB GF2: (5′-GTC TTG TAA TTG GAA TGA TGG-3′) BAB GR2: (5′-CCA AAG ACT TTG ATT TCT CTC-3′)	18S rRNA	559	52	[81]
*B. burgdorferi* s.l	M1: (5′-ACG ATG CAC ACT TGG TGT TAA-3′) M2: (5′-TCC GAC TTA TCA CCG GCA GTC A-3′)	16S rRNA	357	50	[82]
*A. phagocytohilum*	gE9f: (5′-AACGGATTATTCTTTATAGCTTGCT-3′) ge2: (5′-GGCAGTATTAAAAGCAGCTCCAGG-3′)	16S rRNA	546	55	[83]

The table includes the primer sequences, target genes, amplicon sizes, and references for each primer pair. These primers were used to amplify specific regions of the *A. phagocytophilum*, *B. burgdorferi* s.l., *E. canis*, and *Babesia* spp. genomes in the samples collected

## Results

### Tick presence

**Fig. 1:**
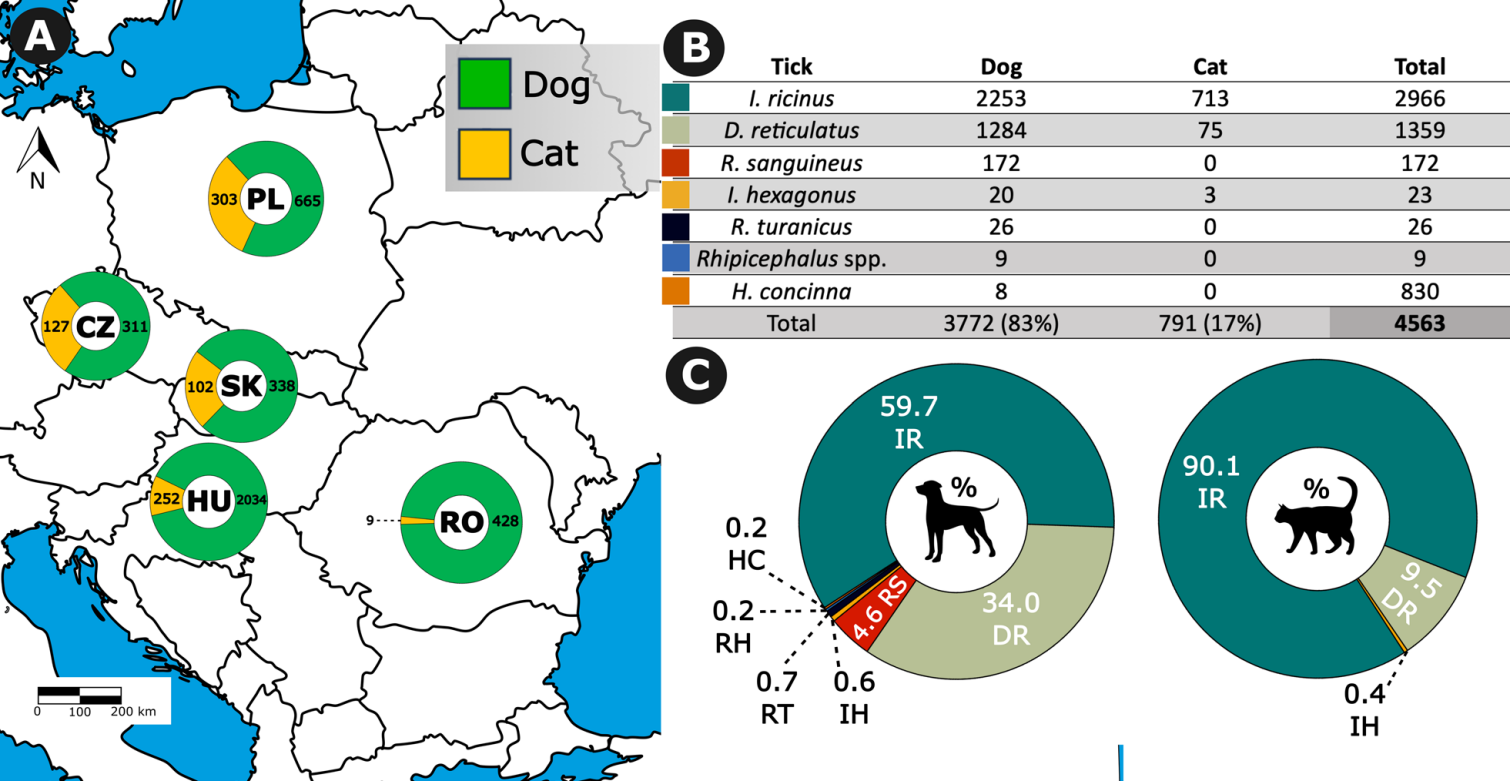
**A** Geographic distribution and presence of total number of ticks in dogs and cats in the Czech Republic (CZ), Slovakia (SK), Poland (PL), Hungary (HU), and Romania (RO). **B** The table presents the number of tick species found in dogs and cats. **C** Combined tick species prevalence in dogs and cats in all countries expressed as percentages. *I. ricinus*, IR; *D. reticulatus*, DR; *R. sanguineus*, RS; *R. turanicus*, RT; *Rhipicephalus* spp., RH; *I. hexagonus*, IH; *H. concinna*, HC

**Fig. 2:**
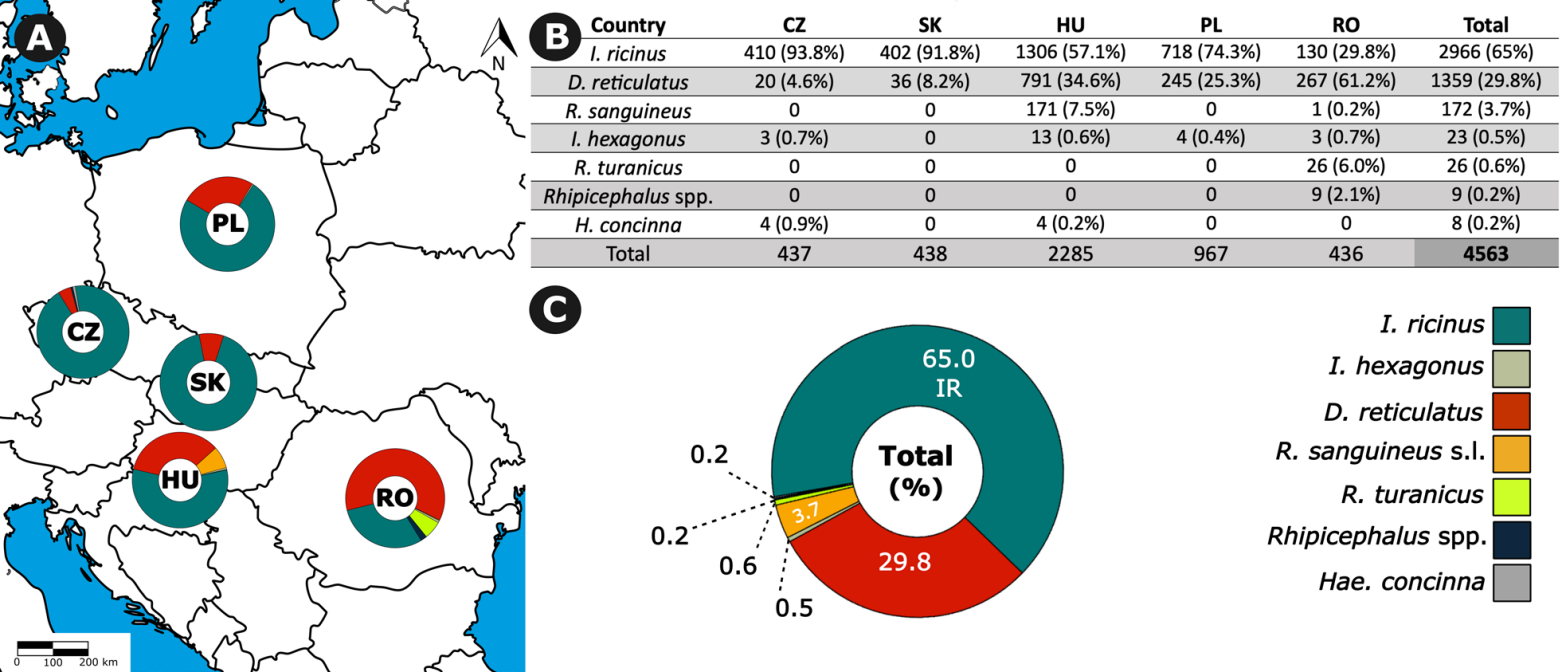
The prevalence of tick species collected from the Czech Republic (CZ), Slovakia (SK), Hungary (HU), Poland (PL), and Romania (RO). **A** The map illustrates the total number of ticks and the distribution of various tick species across each country based on samples obtained from veterinary clinics during the study period. **B** The table presents the number of tick species collected in each country. **C** Combined tick species prevalence in all countries expressed as percentages

In total, 74 veterinary clinics submitted 4563 ticks from dogs and cats; 3772 ticks were collected from dogs, and 791 ticks were collected from cats (Fig. 1). Most of the samples were from Hungary (2285), followed by Poland (967), Slovakia (438), Czech Republic (437), and Romania (436). Of 4563 identified ticks, 215 were immature stages (8 larvae and 207 nymphs), and 4348 were adults. Of 215 immature stages, the majority belonged to the genus *Ixodes* (7 larvae and 192 nymphs), followed by *Rhipicephalus* (9 nymphs) and *Dermacentor* (1 larvae and 6 nymphs). Of 4348 adults, 3470 were identified as females (79.8%) and 878 as males (20.2%). Morphological identification showed that the majority of ticks were *I. ricinus* (2966), followed by *D. reticulatus* (1359), *R. sanguineus* (172), *R. turanicus* (26), *I. hexagonus* (23), unidentified *Rhipicephalus* nymphs (9), and *H. concinna* (8), see Fig. 2.

**Fig. 3:**
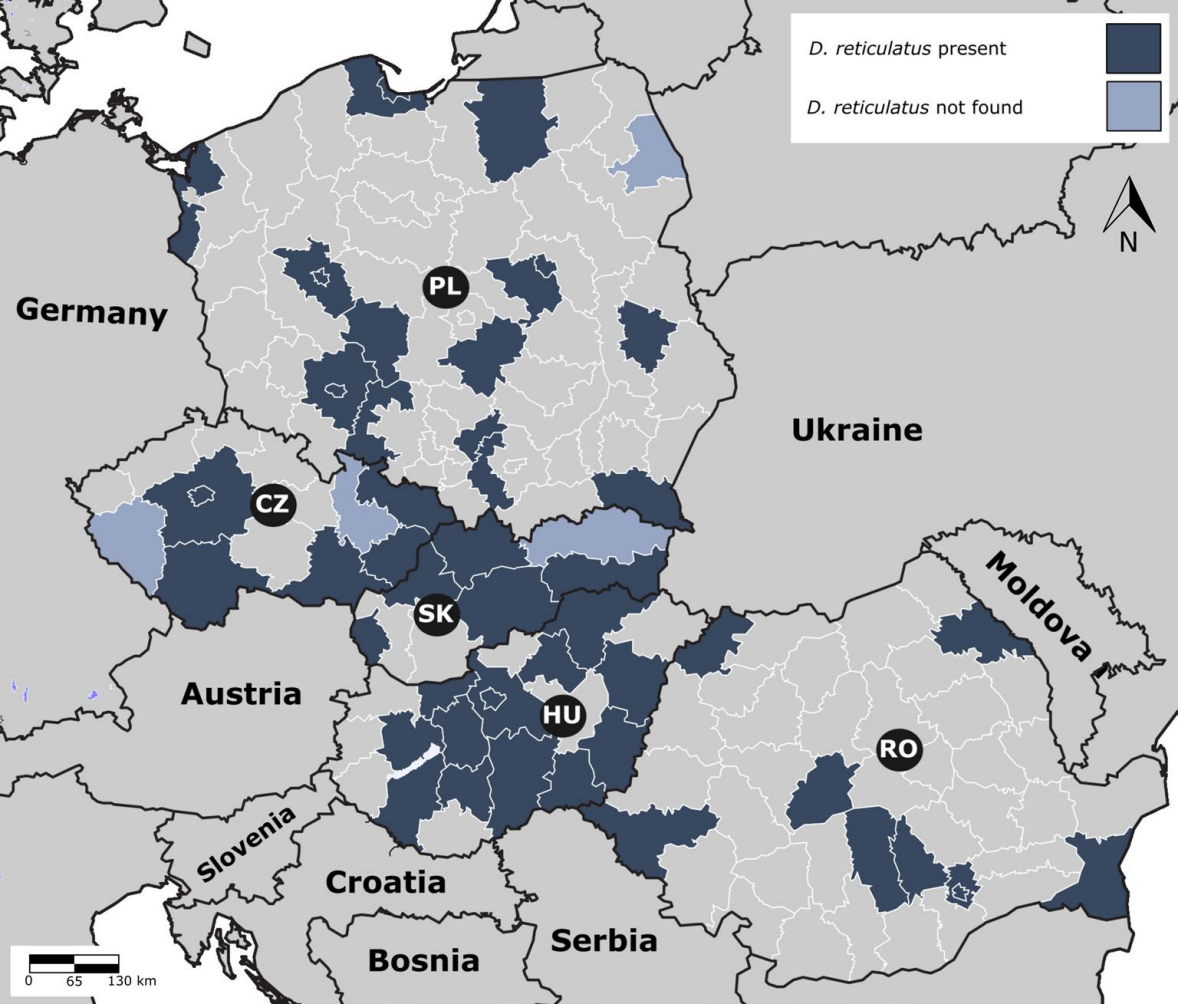
Regions where *D. reticulatus* ticks were found in the Czech Republic (CZ), Slovakia (SK), Hungary (HU), Poland (PL), and Romania (RO). The map illustrates the varying levels of *D. reticulatus* presence in each country based on tick submissions from veterinary clinics. This data reveals regional variations in tick population density

Two tick species, *I. ricinus* and *D. reticulatus*, were most prevalent, in 65.0% and 29.8% of ticks from studied dogs and cats, respectively. *I. ricinus* was dominant in the sample sets in all countries except Romania, where *D. reticulatus* was the most prevalent tick species. The distribution of *D. reticulatus* deserves closer attention regarding its vectorial role for *B. canis*. We received ticks from 51 regions in 5 countries; *D. reticulatus* was collected in 47 (92.2%) regions, while only in 4 (7.8%) regions *D. reticulatus* was not found. Of the regions where *D. reticulatus* was not collected, two were in the Czech Republic, while the remaining were in Slovakia and Poland, with one region in each of these countries. Conversely, *D. reticulatus* was collected in all the sampling regions in Hungary and Romania (Fig. 3).

### Seasonal pattern of the tick distribution

**Fig. 4:**
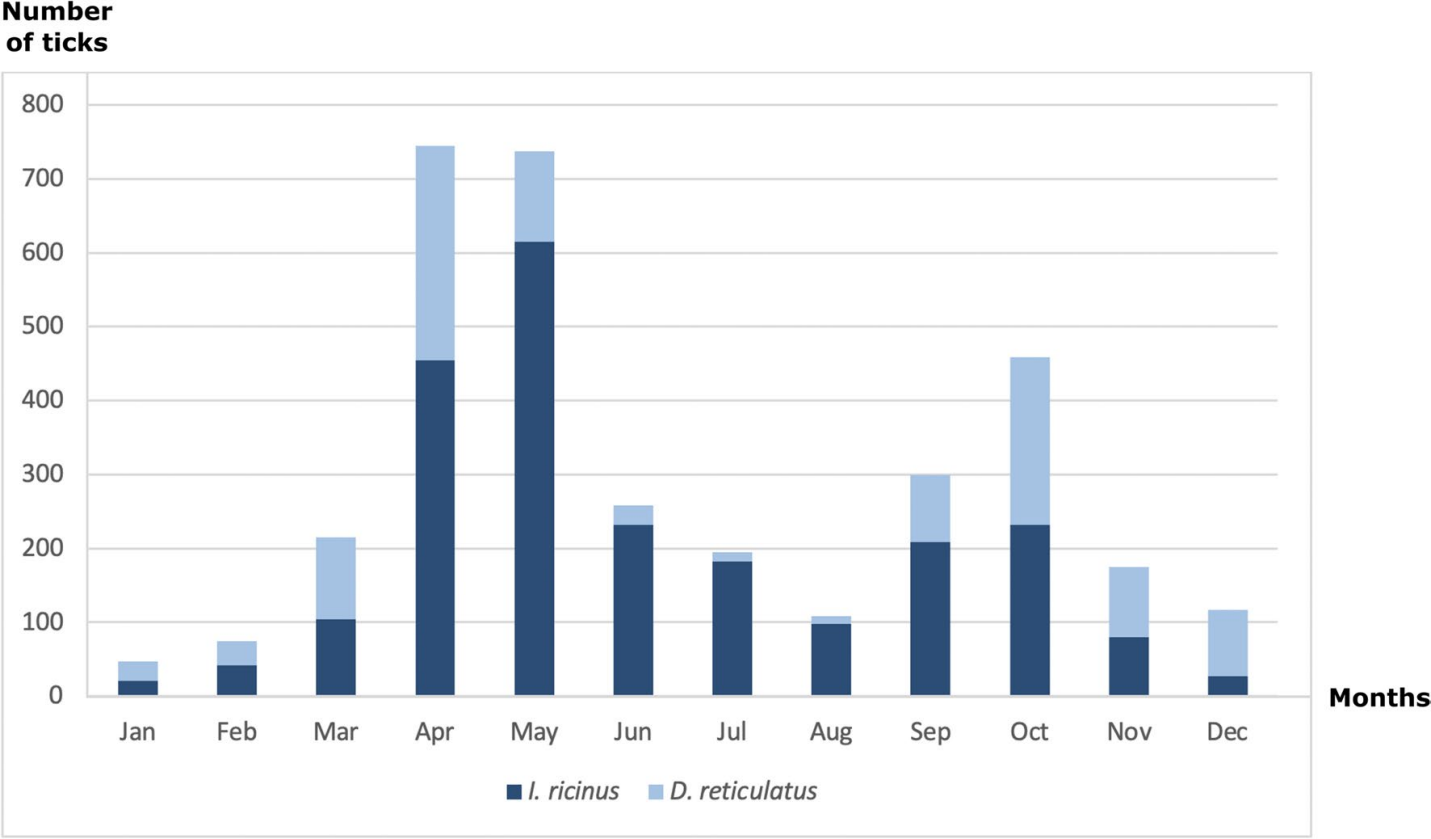
The number of *I. ricinus* and *D. reticulatus* ticks collected by veterinarians on dogs and cats divided into months during the study period from January 2020 to December 2022 in the Czech Republic, Slovakia, Hungary, Poland, and Romania. The *x*-axis illustrates the monthly distribution of collected ticks, while the *y*-axis represents the corresponding quantity of these ticks

The collection date was available for all the collected ticks during the collection period (January 2020–December 2022). The monthly tick occurrence data analysis demonstrates a clear seasonality with peaks in April (in the first half of the year) and October (in the second half of the year) for *D. reticulatus* (Fig. 4). *I. ricinus* also shows an obvious seasonality pattern with peaks in May (in the first half of the year) followed by a second peak in October (in the second half of the year) (Fig. 4). In this study, the infestation seasonal pattern was similar for both dogs and cats.

### Detected pathogens

Of 1085 tested ticks for pathogens, 315 (29.0%) were found with at least one of the tested pathogens. Among the tested tick species, *I. ricinus* was the most infected species (33.0%), followed by *D. reticulatus* (20.7%), while others were negative for any of the pathogens (Table 2).

**Table 2:** Prevalence of *A. phagocytophilum*, *B. burgdorferi* s.l., *E. canis*, and *Babesia* spp. in *I. ricinus*, *D. reticulatus*, and *I. hexagonus* ticks collected from dogs and cats in the Czech Republic, Slovakia, Hungary, Poland, and Romania

Tick (number of tested samples)	*B. burgdorferi*s.l.	*E. canis*	*A. phagocytophilum*	*Babesia* spp.
*I. ricinus* (741)	72 (9.7%)	5 (0.7%)	160 (21.5%)	8 (1.1%)
*D. reticulatus* (338)	4 (1.2%)	0 (0%)	3 (0.9%)	63 (18.6%)
*I. hexagonus* (6)	0 (0%)	0 (0%)	0 (0%)	0 (0%)
Total (1085)	76 (7%)	5 (0.5%)	163 (15%)	71 (6.5%)

*A. phagocytophilum* was the most prevalent pathogen in the tested samples, detected in 15.0% of the tested samples. It was predominantly found in *I. ricinus* (98.0%), with a smaller presence in *D. reticulatus* (2.0%). Ticks collected in Poland had the highest prevalence in comparison with other countries (26.2%), followed by the Czech Republic (17.7%), Slovakia (14.8%), Hungary (12.5%), and Romania (8.1%) (Table 3).

**Table 3:** The number of *A. phagocytophilum*, *B. burgdorferi* s.l., *E. canis*, and *Babesia* spp. positive ticks and prevalence in ticks collected from the Czech Republic (CZ), Slovakia (SK), Hungary (HU), Poland (PL), and Romania (RO). Prevalences marked in bold font represent the highest value for each pathogen

Country (number of tested samples)	*B. burgdorferi*s.l.	*E. canis*	*A. phagocytophilum*	*Babesia* spp.
CZ (339)	32 (9.4%)	1 (0.3%)	60 (17.7%)	6 (1.8%)
SK (108)	6 (5.6%)	0 (0%)	16 (14.8%)	5 (4.6%)
PL (114)	**15 (13.1%)**	**1 (0.9%)**	**30 (26.2%)**	10 (8.8%)
HU (326)	18 (5.5%)	2 (0.6%)	41 (12.5%)	31 (9.4%)
RO (198)	5 (2.5%)	1 (0.5%)	16 (8.1%)	**19 (9.6%)**
Total (1085)	76 (7%)	5 (0.5%)	163 (15%)	71 (6.5%)

*B. burgdorferi* s.l. was the second most prevalent pathogen in the tested samples, detected in 7.0% of ticks. *I. ricinus* showed the highest positivity (95.0%), followed by *D. reticulatus* (5.0%). Ticks originating in Poland had the highest positivity of *B. burgdorferi* s.l. (13.1%), followed by the Czech Republic (9.4%), Slovakia (5.6%), Hungary (5.5%), and Romania (2.5%).

*E. canis* was the pathogen with the lowest prevalence in the tested samples, detected in only 0.5%. *I. ricinus* was the only tick positive for this pathogen. Only 0.7% of the tested *I. ricinus* ticks were positive for *E. canis*. Samples collected in Poland had the most positivity (0.9%) for *E. canis*; however, the difference in positivity between the five countries was relatively small (0.3–0.9%). Only Slovakian samples were negative for *E. canis* (Table 3).

*Babesia* species were detected in 6.5% of the tested samples. *D. reticulatus* was the primary carrier (89.0%), followed by *I. ricinus* (11.0%). *B. canis* was the most detected species of *Babesia* (91.5%). Sequences belonging to the *B. microti* group and unclassified sequences assigned as *Babesia* sp. were each detected in 4.2%. *B. canis* was detected mainly in *D. reticulatus* (97.0%), with a much smaller presence in *I. ricinus* (3.0%). The *B. microti* group and *Babesia* sp. were detected only in *I. ricinus* (100%). Considering the prevalence in the whole sample set, *Babesia* spp. were more commonly detected in ticks collected in Romania, Poland, and Hungary than in those from the Czech Republic and Slovakia (Table 3).

## Discussion

### Tick species prevalence

In line with previous studies [1, 33, 34], the present study identified *I. ricinus*, *R. sanguineus* s.l., *D. reticulatus*, and *I. hexagonus* as the dominant ticks infesting dogs and cats in Poland, the Czech Republic, Slovakia, Hungary, and Romania. Not surprisingly, the occurrence and prevalence of individual tick species differed across sampled countries, reflecting the major distribution pattern of species found in Europe [35]. In general, the distribution and population densities of tick species within a biogeographic zone are influenced by several factors, including the biotope, which can be more or less favorable to tick development (ecological preferences), the availability of preferred hosts (trophic preferences), and climatic conditions [36, 37]. Dynamic changes in the distribution of tick species (and associated pathogens), driven by socio-economic development, animal movement, and environmental and climatic changes [38], can be exemplified in Europe using *R. sanguineus* s.l. and *D. reticulatus*.

In Europe, *R. sanguineus* s.l., a Mediterranean tick species, is recognized for its gradual focal dispersal into temperate regions, which is evidenced by a growing number of findings in northern countries, such as the UK, Ireland, Denmark, Germany, and Sweden [4–6, 39]. The northward expansion of this thermophilic tick, followed by its increased presence and the emergence of transmitted pathogens in originally non-endemic regions, is attributed to global climate changes combined with domestic dog movement [38, 40–42]. In our study, this tick was only collected by veterinary practitioners in Romania and Hungary. While Romania is a traditionally known as an area of its presence [1, 43, 44], our records from Hungary confirmed a previously reported isolated population of this tick in the northeastern part of Tolna County [6, 34]. Moreover, our study confirms that countries north of Hungary are still free from regular infestations of *R. sanguineus.*

*D. reticulatus* is more prevalent in the temperate and continental zones, particularly in lowland and humid ones [45–48]. This tick, originally considered a species with a discontinuous geographic range, is now rapidly spreading through temperate Europe [19, 45, 49–58]. Factors contributing to this expansion involve its climate adaptability and host availability. Also, in the case of this tick, domestic dogs, as dominant carnivores in the whole of Europe, play a pivotal role in its expanding distribution [32, 54, 59]. In line with other studies [28, 60], the results obtained in this study demonstrated that *D. reticulatus* mainly infests dogs (34.0%) compared with cats (9.5%). The data on the distribution of *D. reticulatus* in our study confirm its importance across the studied region, including Czech Republic, where its broad distribution emerged only recently [19]. Surprisingly, *D. reticulatus* was not found in the northeast region of Poland, while this tick has been found in vegetation [61]. However, the collection in the present study was done on more urban animals, and the infestation could not be observed due to lower exposure. The presence of *D. reticulatus* was confirmed for a large area of Poland. However, a strip of land along the northwest–southeast axis (~151,000 km^2^) extending from the northwest to mountain areas with foothills in the south, including Podkarpackie voivodeship, was presented [54]. Therefore, the lack of infestations seen in the northeast of Slovakia observed in the present study confirms the tick distribution expansion shown before.

### Dog and cat infestations

In this study, the majority of the collected ticks were found on dogs. The study design does not allow for prevalence assessment, as the number of dogs/cats without ticks or the total number of animals referred to collaborating clinics are unavailable. Contrary to our findings, other similar studies showed that the number of ticks found in cats was only slightly lower (up to 12.0%) than that in dogs [28, 62]. These discrepancies may be attributed to local factors, such as differences in pet care practices, the frequency of veterinary clinic visits for dogs versus cats (e.g., in Romania), or variations in outdoor exposure, with dogs generally spending more time in tick-suitable habitats [63]. Cats are meticulous groomers and are likely to remove ticks themselves before they have time to attach and transmit pathogens, while dogs are less efficient in self-grooming [64]. Different tick prevalence on both hosts, perhaps also reflects the host preferences of the tick species involved. Tick species such as *R. sanguineus* and *D. reticulatus* show a higher dog infestation rate. At the same time, cats are predominantly affected by *I. ricinus* [28], and this is confirmed by the present study.

### Seasonality of tick infestations

Various ticks exhibit distinct seasonal patterns, depending on climate seasonality combined with geographical latitude and altitude above sea level, and these patterns have recently been impacted by climate change [15, 65]. In the present study, the two dominant tick species, *I. ricinus* and *D. reticulatus*, were collected throughout all 12 months of the year, though there was a noticeable drop in the number of infestation events from December to February compared with other months. The occurrence of both species followed a similar bimodal pattern, with high occurrences on dogs and cats seen in late March, April, and May, with a gradual decline until the end of August; then, the second peak was observed in September–October. Similar patterns of *I. ricinus* and *D. reticulatus* infestations were observed on dogs and cats in Germany [28]. The spring maximum of *I. ricinus* was observed in our study 1 month earlier compared with studies in the UK [66, 67], while it was similar to a study in Germany [28]. The seasonality of *D. reticulatus* infestations observed in the present study is consistent with previous studies [19, 68].

### Pathogens in ticks

The prevalence of *A. phagocytophilum* and *Borrelia burgdorferi* s.l. in our study reflects the dominant role of *I. ricinus* in its circulation. Finding *A. phagocytophilum* and *B. burgdorferi* s.l. in ~20.0% and 9.0% of *I. ricinus*, respectively, aligns well with the broader European context, where the prevalence of *A. phagocytophilum* in *I. ricinus* varies greatly, ranging from 1.0% to 50.0%, depending on the region and specific study [69–72]. *D. reticulatus* is generally considered a less significant vector of *A. phagocytophilum* and *Borrelia burgdorferi* s.l., with low infection rates across Europe that range from 1.0% to 1.5% [73–75]. However, the detected prevalence in our study should not be disregarded, and this emerging tick species deserves more attention, even though its actual vectorial competence has not yet been proven. Surprisingly, *E. canis* DNA was detected in five (0.7%) *I. ricinus* ticks, even though this bacterium is typically associated with ticks of the *R. sanguineus* complex [70, 76]. Although *E. canis* has been occasionally detected in *I. ricinus*, such occurrences are rare, and this species is considered a minor vector compared with *R. sanguineus* [71]. The presence of *E. canis* in samples from Romania and Hungary can be explained by the possible spatial co-existence (and co-feeding?) of *I. ricinus* and *R. sanguineus*; however, detection of *E. canis* outside of the *R. sanguineus* range in Poland and the Czech Republic deserves attention and is a novel finding of the present study.

The DNA of *Babesia* spp. was found in 6.5% of examined ticks, with *B. canis* dominating (91.5%). The presented data reflect a strong association of *B. canis* with *D. reticulatus* (6.7% to 18.6% prevalence), as concluded in other studies [19, 22, 77, 78]. Conversely, the prevalence of *Babesia* spp. in *I. ricinus* ticks shown in the present study was very low (1.1%) and is similar to other studies [79].

The presented data reveal differences in tick-borne pathogen prevalence across the studied European countries. As discussed above, these differences reflect the composition of tick fauna on dogs and cats in surveyed countries. *I. ricinus* was a dominant tick in the whole sample set (65.0%), as well as in all surveyed countries except Romania (Fig. 2). The tick-borne pathogens transmitted by this tick species are also the most prevalent ones, as obvious in the case of *B. burgdorferi* s.l. and *A. phagocytophilum*, which were most frequently detected in Poland, while the detection rate was lowest in Romania. Similarly, *Babesia* spp. were detected most frequently in Romania (9.6%), followed by Hungary (6.1%) and Poland (6.1%), reflecting the proportion of *D. reticulatus* in the sample sets from these countries.

## Conclusions

The present study provides insights into the species composition and seasonal patterns of ticks infesting dogs and cats in Central Europe. Changes in the geographic distribution of some tick species and associated tick-borne diseases pose a growing risk to both animals and humans in these regions. The data underscore distinct geographical patterns of tick distribution, mirrored into pathogen prevalence and risk of infection, emphasizing the need for targeted surveillance and control measures tailored to each region’s risk profile. We confirmed that *I. ricinus* and *D. reticulatus* are the most prevalent tick species infesting dogs and cats in the studied region, while *R. sanguineus* has been documented mainly in Hungary and in small numbers in Romania. The infestation seasonality has a two-peak pattern for the most prevalent species; however, tick infestations were reported every month of the year, supporting the importance of all-year protection against these parasites. Our findings reinforce the need for proactive measures to protect dogs, cats, and humans in the veterinary and public health sectors.

## Supplementary Information

Supplementary Material 1: The number of pathogens detected in ticks collected from different towns and cities in Hungary, the Czech Republic, Romania, Slovakia, and Hungary. These tables present the distribution of pathogen species identified across various geographical locations, highlighting the prevalence and diversity of tick-borne diseases

## Data Availability

Data supporting the main conclusions of this study are included in the manuscript.

## Abbreviations

CZ: The Czech Republic
PL: Poland
RO: Romania
SK: Slovakia
HU: Hungary
