# Supplementary material for: Diversity of ticks and tick-borne pathogens in ticks removed from dogs and cats: a focus on Poland, Czech Republic, Slovakia, Hungary, and Romania
Source: Parasit Vectors. 2025 Jul 21;18:290. doi: 10.1186/s13071-025-06852-6 (PMC12281808; doi:10.1186/s13071-025-06852-6)
Supplement: Supplementary file 1 — Supplementary Material 1: The number of pathogens detected in ticks collected from different towns and cities in Hungary, the Czech Republic, Romania, Slovakia, and Hungary. These tables present the distribution of pathogen species identified across various geographical locations, highlighting the prevalence and diversity of tick-borne diseases [file 13071_2025_6852_MOESM1_ESM.pdf]

Table S1. Number of pathogens detected in ticks collected from different towns and cities in Hungary. This table presents the distribution of pathogen species identified across various geographical locations, highlighting the prevalence and diversity of tick-borne diseases.

Town	Total tested	*B.burgdorferi s.l.*	*E. canis*	*A. phagocytophilum*	*Babesia* spp.	*B. canis*	*B. microti*	Total positive
Csongrád	**26**	1 (3.8%)	0 (0%)	5 (19.2%)	0 (0%)	1 (3.8%)	0 (0%)	**7 (26.9%)**
Szigetvár	**5**	0 (0%)	0 (0%)	0 (0%)	0 (0%)	2 (40%)	0 (0%)	**2 (40%)**
Jászberény	**16**	0 (0%)	0 (0%)	4 (25%)	0 (0%)	2 (12.5%)	0 (0%)	**6 (37.5%)**
Csurgó	**17**	2 (11.8%)	0 (0%)	2 (11.8%)	0 (0%)	3 (17.6%)	0 (0%)	**7 (41.2%)**
Kecskemét	**37**	0 (0%)	0 (0%)	1 (2.7%)	0 (0%)	2 (5.40%)	0 (0%)	**3 (8.1%)**
Kaposfő	**29**	2 (6.9%)	0 (0%)	1 (3.4%)	0 (0%)	3 (10.3%)	0 (0%)	**6 (20.7%)**
Mátészalka	**20**	1 (5%)	0 (0%)	2 (10%)	0 (0%)	1 (5%)	0 (0%)	**4 (20%)**
Paks	**12**	2 (16.7%)	0 (0%)	2 (16.7%)	0 (0%)	1 (8.3%)	0 (0%)	**5 (41.7%)**
Gyula	**19**	0 (0%)	0 (0%)	6 (31.6%)	0 (0%)	1 (5.3%)	0 (0%)	**7 (36.8%)**
Diósd	**17**	1 (5.9%)	2 (11.8%)	2 (11.8%)	0 (0%)	2 (11.8%)	1 (5.9%)	**8 (47%)**
Balatonfüred	**19**	2 (10.5%)	0 (0%)	4 (21%)	0 (0%)	1 (5.3%)	0 (0%)	**7 (36.8%)**
Mór	**17**	1 (5.9%)	0 (0%)	1 (5.9%)	0 (0%)	1 (5.9%)	0 (0%)	**3 (17.6%)**
Budapest	**20**	1 (5%)	0 (0%)	2 (10%)	0 (0%)	0 (0%)	1 (5%)	**4 (20%)**
Komárom	**15**	0 (0%)	0 (0%)	1 (6.7%)	0 (0%)	3 (20%)	0 (0%)	**4 (26.7%)**
Alsózsolca	**22**	0 (0%)	0 (0%)	0 (0%)	0 (0%)	3 (13.6%)	0 (0%)	**3 (13.6%)**
Győr	**24**	5 (20.8%)	0 (0%)	7 (29.2%)	0 (0%)	0 (0%)	1 (4.2%)	**13 (54.2%)**
Miskolc	**12**	0 (0%)	0 (0%)	1 (8.3%)	0 (0%)	2 (16.7%)	0 (0%)	**3 (25%)**

Table S2. Number of pathogens detected in ticks collected from different towns and cities in the Czech Republic. This table presents the distribution of pathogen species identified across various geographical locations, highlighting the prevalence and diversity of tick-borne diseases.

Town	Total tested	*B.burgdorferi s.l.*	*E. canis*	*A. phagocytophilum*	*Babesia* spp.	*B. canis*	*B. microti*	Total positive
Praha - Rudná	**9**	1 (1.1%)	0 (0%)	2 (22.2%)	0 (0%)	0 (0%)	0 (0%)	**3 (33.3%)**
Plzeň	**22**	1 (4.5%)	0 (0%)	1 (4.5%)	1 (4.5%)	0 (0%)	0 (0%)	**3 (13.6%)**
České Budějovice	**10**	1 (10%)	0 (0%)	2 (20%)	0 (0%)	1 (10%)	0 (0%)	**4 (40%)**
Praha 4	**6**	0 (0%)	0 (0%)	0 (0%)	0 (0%)	0 (0%)	0 (0%)	**0 (0%)**
Olomouc	**13**	0 (0%)	0 (0%)	0 (0%)	1 (7.7%)	0 (0%)	0 (0%)	**1 (7.7%)**
Vyškov	**7**	0 (0%)	0 (0%)	0 (0%)	0 (0%)	0 (0%)	0 (0%)	**0 (0%)**
Velké Pavlovice	**49**	2 (4.1%)	1 (2.0%)	11 (22.4%)	1 (2%)	0 (0%)	0 (0%)	**15 (30.6%)**
Břeclav	**4**	1 (25%)	0 (0%)	1 (25%)	0 (0%)	0 (0%)	0 (0%)	**2 (50%)**
Uherské Hradiště	**1**	0 (0%)	0 (0%)	0 (0%)	0 (0%)	0 (0%)	0 (0%)	**0 (0%)**
Uherský Brod	**1**	0 (0%)	0 (0%)	0 (0%)	0 (0%)	0 (0%)	0 (0%)	**0 (0%)**
Hodonín	**16**	6 (37.5%)	0 (0%)	4 (25%)	0 (0%)	0 (0%)	0 (0%)	**10 (62.5%)**
Veselí na Moravě	**96**	13 (13.5%)	0 (0%)	19 (19.8%)	0 (0%)	2 (2.1%)	0 (0%)	**34 (35.4%)**
Mníšek pdo Brdy	**8**	2 (25%)	0 (0%)	3 (37.5%)	0 (0%)	0 (0%)	0 (0%)	**5 (62.5%)**
Praha - Zbraslav	**26**	2 (7.7%)	0 (0%)	4 (15.4%)	0 (0%)	0 (0%)	0 (0%)	**6 (23.1%)**
Praha 9	**5**	0 (0%)	0 (0%)	1 (20%)	0 (0%)	0 (0%)	0 (0%)	**1 (20%)**
Znojmo	**9**	1 (11.1%)	0 (0%)	2 (22.2%)	0 (0%)	0 (0%)	0 (0%)	**3 (33.3%)**
Kroměříž	**16**	1 (6.2%)	0 (0%)	3 (18.8%)	0 (0%)	0 (0%)	0 (0%)	**4 (25%)**
Český Těšín	**39**	1 (2.6%)	0 (0%)	6 (15.4%)	0 (0%)	0 (0%)	0 (0%)	**7 (17.9%)**

Table S3. Number of pathogens detected in ticks collected from different towns and cities in Romania. This table presents the distribution of pathogen species identified across various geographical locations, highlighting the prevalence and diversity of tick-borne diseases.

Town	Total tested	*B.burgdorferi s.l.*	*E. canis*	*A. phagocytophilum*	*Babesia* spp.	*B. canis*	*B. microti*	Total positive
Satu Mare	**46**	1 (2.2%)	0 (0%)	1 (2.2%)	0 (0%)	4 (8.7%)	0 (0%)	**6 (13%)**
București	**9**	0 (0%)	0 (0%)	1 (11.1%)	0 (0%)	0 (0%)	0 (0%)	**1 (11.1%)**
Iași	**39**	1 (2.6%)	1 (2.6%)	2 (5.1%)	0 (0%)	2 (5.1%)	0 (0%)	**6 (15.4%)**
Dumbrăvița	**18**	1 (5.5%)	0 (0%)	1 (5.5%)	0 (0%)	0 (0%)	0 (0%)	**2 (11.1%)**
Dancu	**4**	0 (0%)	0 (0%)	0 (0%)	0 (0%)	1 (2%)	0 (0%)	**1 (25%)**
Sibiu	**64**	1 (1.6%)	0 (0%)	8 (12.5%)	0 (0%)	9 (14.1%)	0 (0%)	**18 (28.1%)**
Pitești	**1**	0 (0%)	0 (0%)	1 (100%)	0 (0%)	0 (0%)	0 (0%)	**1 (100%)**
București	**17**	1 (5.9%)	0 (0%)	1 (5.9%)	0 (0%)	3 (17.6%)	0 (0%)	**5 (29.4%)**

Table S4. Number of pathogens detected in ticks collected from different towns and cities in Slovakia. This table presents the distribution of pathogen species identified across various geographical locations, highlighting the prevalence and diversity of tick-borne diseases.

Town	Total tested	*B.burgdorferi s.l.*	*E. canis*	*A. phagocytophilum*	*Babesia* spp.	*B. canis*	*B. microti*	Total positive
Lučenec	**1**	0 (0%)	0 (0%)	0 (0%)	0 (0%)	0 (0%)	0 (0%)	**0 (0%)**
Hnúšta	**3**	0 (0%)	0 (0%)	1 (33.3%)	0 (0%)	0 (0%)	0 (0%)	**1 (33.3%)**
Mad	**28**	4 (14.3%)	0 (0%)	8 (28.6%)	0 (0%)	2 (7.1%)	0 (0%)	**14 (50%)**
Košice	**16**	1 (6.2%)	0 (0%)	0 (0%)	0 (0%)	2 (12.5%)	0 (0%)	**3 (18.7%)**
Myjava	**59**	1 (1.7%)	0 (0%)	7 (11.9%)	0 (0%)	1 (1.7%)	0 (0%)	**9 (15.2%)**

Table S5. Number of pathogens detected in ticks collected from different towns and cities in Poland. This table presents the distribution of pathogen species identified across various geographical locations, highlighting the prevalence and diversity of tick-borne diseases.

Town	Total tested	*B.burgdorferi s.l.*	*E. canis*	*A. phagocytophilum*	*Babesia spp.*	*B. canis*	*B. microti*	Total positive
Konstancin Jeziorna	**6**	1 (16.7%)	0 (0%)	4 (66.7%)	0 (0%)	1 (16.7%)	0 (0%)	**6 (100%)**
Kielce	**5**	0 (0%)	0 (0%)	0 (0%)	0 (0%)	1 (20%)	0 (0%)	**1 (20%)**
Jaworzno	**10**	2 (20%)	0 (0%)	3 (30%)	0 (0%)	0 (0%)	0 (0%)	**5 (50%)**
Szczecin	**9**	1 (11.1%)	0 (0%)	2 (22.2%)	0 (0%)	2 (22.2%)	0 (0%)	**5 (55.5%)**
Lublin	**6**	0 (0%)	0 (0%)	1 (16.7%)	0 (0%)	2 (33.3%)	0 (0%)	**3 (50%)**
Rzeszów	**17**	2 (11.8%)	1 (5.9%)	9 (52.9%)	0 (0%)	1 (5.9%)	0 (0%)	**13 (76.5%)**
Sochaczew	**4**	0 (0%)	0 (0%)	0 (0%)	0 (0%)	1 (25%)	0 (0%)	**1 (25%)**
Ostrów Wielkopolski	**4**	2 (50%)	0 (0%)	1 (25%)	0 (0%)	0 (0%)	0 (0%)	**3 (75%)**
Poznań	**10**	2 (20%)	0 (0%)	4 (40%)	0 (0%)	0 (0%)	0 (0%)	**6 (60%)**
Olsztyn	**14**	0 (0%)	0 (0%)	1 (7.1%)	0 (0%)	2 (14.3%)	0 (0%)	**3 (21.4%)**
Wrocław	**1**	0 (0%)	0 (0%)	0 (0%)	0 (0%)	0 (0%)	0 (0%)	**0 (0%)**
Brzeg	**2**	0 (0%)	0 (0%)	0 (0%)	0 (0%)	0 (0%)	0 (0%)	**0 (0%)**
Czarna Białostocka	**11**	1 (9.1%)	0 (0%)	2 (18.2%)	0 (0%)	0 (0%)	0 (0%)	**3 (27.3%)**
Gdańsk	**1**	0 (0%)	0 (0%)	0 (0%)	0 (0%)	0 (0%)	0 (0%)	**0 (0%)**
